# Genetic diversity of *Plasmodium vivax* and *Plasmodium falciparum* lactate dehydrogenases in Myanmar isolates

**DOI:** 10.1186/s12936-020-3134-y

**Published:** 2020-02-04

**Authors:** Jinyoung Lee, Tae Im Kim, Hương Giang Lê, Won Gi Yoo, Jung-Mi Kang, Seong-Kyu Ahn, Moe Kyaw Myint, Khin Lin, Tong-Soo Kim, Byoung-Kuk Na

**Affiliations:** 1 Department of Tropical Medicine and Inha Research Institute for Medical Science, Inha University School of Medicine, Incheon, Republic of Korea; 2 Department of Parasitology and Tropical Medicine, and Institute of Health Sciences, Gyeongsang National University College of Medicine, Jinju, 52727 Republic of Korea; 3 BK21Plus Team for Anti-aging Biotechnology and Industry, Department of Convergence Medical Science, Gyeongsang National University, Jinju, 52727 Republic of Korea; 4 Department of Medical Environmental Biology, Chung-Ang University College of Medicine, Seoul, 06974 Republic of Korea; 5 Department of Medical Research Pyin Oo Lwin Branch, Pyin Oo Lwin, Myanmar; 6 Present Address: Planning and Management Division, Nakdonggang National Institute of Biological Resources, Sangju, 37242 Republic of Korea

**Keywords:** *Plasmodium vivax*, *Plasmodium falciparum*, Lactate dehydrogenase, Myanmar, Genetic diversity

## Abstract

**Background:**

*Plasmodium* lactate dehydrogenase (pLDH) is a major target in diagnosing the erythrocytic stage of malaria parasites because it is highly expressed during blood-stage parasites and is distinguished from human LDH. Rapid diagnostic tests (RDTs) for malaria use pLDH as a target antigen; however, genetic variations in pLDH within the natural population threaten the efficacy of pLDH-based RDTs.

**Methods:**

Genetic polymorphisms of *Plasmodium vivax* LDH (PvLDH) and *Plasmodium falciparum* LDH (PfLDH) in Myanmar isolates were analysed by nucleotide sequencing analysis. Genetic polymorphisms and the natural selection of PvLDH and PfLDH were analysed using DNASTAR, MEGA6, and DnaSP ver. 5.10.00 programs. The genetic diversity and natural selection of global PvLDH and PfLDH were also analysed. The haplotype network of global PvLDH and PfLDH was constructed using NETWORK ver. 5.0.0.3. Three-dimensional structures of PvLDH and PfLDH were built with YASARA Structure ver. 18.4.24 and the impact of mutations on structural change and stability was evaluated with SDM ver. 2, CUPSAT and MAESTROweb.

**Results:**

Forty-nine PvLDH and 52 PfLDH sequences were obtained from Myanmar *P. vivax* and *P. falciparum* isolates. Non-synonymous nucleotide substitutions resulting in amino acid changes were identified in both Myanmar PvLDH and PfLDH. Amino acid changes were also identified in the global PvLDH and PfLDH populations, but they did not produce structural alterations in either protein. Low genetic diversity was observed in global PvLDH and PfLDH, which may be maintained by a strong purifying selection.

**Conclusion:**

This study extends knowledge for genetic diversity and natural selection of global PvLDH and PfLDH. Although amino acid changes were observed in global PvLDH and PfLDH, they did not alter the conformational structures of the proteins. These suggest that PvLDH and PfLDH are genetically well-conserved in global populations, which indicates that they are suitable antigens for diagnostic purpose and attractive targets for drug development.

## Background

Malaria is an acute febrile infectious disease caused by *Plasmodium* parasites and is widely endemic in tropical and subtropical areas. Although the global incidence and mortality of malaria have decreased in recent years, malaria is still a major public health concern with an estimated 219 million cases causing 435,000 deaths in 2017 [1]. Despite advances in malaria diagnostic methods, microscopic examination of blood smear still remains the gold standard for malaria diagnosis [2]. This method can identify the *Plasmodium* species and quantify the parasitaemia level at a low cost [1]. However, it is laborious, time-consuming, and requires well-trained and highly qualified technicians. Also, misdiagnoses and incorrect species identification can occur in cases of low parasitaemia, leading to incorrect treatment [3]. To overcome these disadvantages, several alternative methods for malaria diagnosis have been developed.

Rapid diagnostic tests (RDTs) that detect plasmodial antigens circulating in the blood during the infection are the most powerful alternatives to microscopy, especially in conditions where microscopic examination is not feasible [4–6]. RDTs have been used as reliable, massive screening tools in malaria-endemic areas. However, several important issues including diagnostic accuracy, high cost, and reliability in unfavorable field conditions plague RDTs. Several plasmodial antigens including histidine-rich protein 2 (pHRP-2), plasmodial aldolase, and plasmodial lactate dehydrogenase (pLDH) have been used in RDTs. Among them, pHRP-2, especially *P. falciparum* HRP-2 (PfHRP-2), has been used in numerous RDTs because combination (or combo) RDTs can target antigens for *P. falciparum* (PfHRP-2) and non-falciparum (or pan-specific; pLDH), simultaneously. However, concerns for HRP-2-based RDTs, especially for *P. falciparum*, have increased in recent years due to false-negative results caused by the deletion of the target gene in malaria parasites [7–12]. Therefore, many manufactures are developing RDTs that can replace PfHRP-2-based RDTs.

pLDH is an enzyme essential to a *Plasmodium* parasite’s survival by mediating parasite’s anaerobic glycolysis, and it is expressed at high levels during the blood stage [13, 14]. Moreover, the level of pLDH in the blood is directly correlated to the parasitaemia level. Due to these advantages, pLDH has been widely examined as a promising diagnostic antigen and has been adopted in many RDTs for malaria. However, few studies have examined pLDH genetic polymorphisms in *Plasmodium* isolates and the potential influence pLDH genetic variations have on the diagnostic performance of pLDH-based RDTs [15–17]. In this study, the genetic diversity of *P. falciparum* LDH (PfLDH) and *P. vivax* LDH (PvLDH) in Myanmar parasite isolates was investigated. Moreover, the genetic diversity of global PfLDH and PvLDH populations was also analysed to better understand the genetic structures of the two genes in global *P. falciparum* and *P. vivax* populations.

## Methods

### Blood samples and ethics

The blood samples used in this study were obtained from *P. vivax* and *P. falciparum* infected patients with symptomatic uncomplicated cases who resided in towns and villages located in Naung Cho and Pyin Oo Lwin townships in Upper Myanmar in 2015. Malaria infection was confirmed by Giemsa-stained thick and thin blood smear examinations. Prior to treatment, 2 ml of venous blood was collected from each confirmed *P. falciparum* and *P. vivax* infected patients and placed into ethylenediaminetetraacetic acid (EDTA) tubes for further molecular analysis. All *P. falciparum* and *P. vivax* positive samples were further confirmed with polymerase chain reaction (PCR) targeting the 18S ribosomal RNA (rRNA) gene [18, 19]. The use of blood samples in this study was approved by the Ministry of Health, Myanmar (97/Ethics 2015) and by the Biomedical Research Ethics Review Board of Inha University School of Medicine, Republic of Korea (INHA 15-013). Written consent was obtained from each individual prior to blood collection.

### Amplification and sequencing analysis of the PvLDH and PfLDH

The genomic DNA of parasite was extracted from 200 µl of whole blood using the QIAamp DNA Blood Kit (Qiagen, Hilden, Germany) following the manufacturer’s instructions. PvLDH and PfLDH were amplified using PCR with primer sets described previous [15]. Briefly, the forward and reverse primers used to amplify PvLDH were 5′-ATGACGCCGAAACCCAAAAT-3′ and 5′-ACCTTTAAATGAGCGCCTTCAT-3′. The primers for PfLDH amplification was 5′-AGATGGCACCAAAAGCAAAAAT-3′ and 5′-ACCTTTAAGCTAATGCCTTCAT-3′. The thermal cycling parameters for the PCR were as follows: denaturation at 94 °C for 5 min; 30 cycles of 94 °C for 1 min, annealing at 56 °C for 1 min, and extension at 72 °C for 1 min; and a final extension at 72 °C for 10 min. To minimize nucleotide mismatches during amplification, Ex *Taq* DNA polymerase (Takara, Otsu, Japan), which has proofreading activity, was used in all PCR procedures. Each PCR product was analysed by electrophoresis on 1% agarose gels. The resulting PCR product was purified from the gel and cloned into the T&A cloning vector (Real Biotech Corporation, Banqiao City, Taiwan). Each ligation mixture was transformed into *Escherichia coli* DH5α competent cells and positive clones with the appropriate insert were screened by colony PCR. The nucleotide sequences of the cloned PCR product were analysed by automatic sequencing using the BigDye™ Terminator Cycle Sequencing Kit (Applied Biosystems, Foster City, CA, USA) on an ABI3730XL DNA analyzer (Applied Biosystems) with M13 forward and reverse primers. At least two clones from each isolate were sequenced to ensure sequencing accuracy, and some isolates underwent three or fourfold sequence coverage to confirm the existence of rare polymorphisms. The nucleotide sequences reported in this study have been deposited in the GenBank database under the accession numbers KU869730–KU869766 and KX885908–KX885922 for PfLDH and KU895512–KU895548 and KX885923–KX885934 for PvLDH.

### Nucleotide sequence polymorphism analysis and neutrality test

The nucleotide and deduced amino acid sequences of Myanmar PfLDH and PvLDH were analysed using EditSeq and SeqMan programs in the DNASTAR software package (DNASTAR, Madison, WI, USA). Nucleotide sequence polymorphism analysis was conducted for the 52 PfLDH and 49 PvLDH sequences. The number of segregating sites (S), the number of haplotypes (H), haplotype diversity (Hd), nucleotide diversity (π), and the average number of pairwise nucleotide differences within the population (*K*) were estimated using DnaSP ver. 5.10.00. [20]. The π was calculated to estimate the stepwise diversity throughout Myanmar PfLDH and PvLDH based on a sliding window of 50 base pairs (bp) with a step size of 10 bp. The values of synonymous (dS) and non-synonymous (dN) substitutions were estimated and were compared using the Z-test (*P* < 0.05) in the MEGA4 program [21] using Nei and Gojobori’s method [22] with the Jukes and Cantor correction. The Tajima’s D value [23] and Fu and Li’s D and F statistics [24] were analysed using the DnaSP ver. 5.10.00 to evaluate the neutral theory of evolution [20]. Sliding window plot analysis was also performed to analyse the step-wide value of Tajima’s D throughout the Myanmar PfLDH and PvLDH based on a sliding window of 100 bp with a step size of 25 bp. The recombination parameter (R), which included the effective population size and probability of recombination between adjacent nucleotides per generation, and the minimum number of recombination events (Rm) were determined using DnaSP ver. 5.10.00 [20].

### Genetic diversity of PvLDH and PfLDH in global *Plasmodium falciparum* and *Plasmodium vivax* populations

The genetic diversity of global PfLDH and PvLDH were analysed. The publicly available sequences of global PvLDH and PfLDH were used in this study (Additional files 1 and 2: Tables S1 and S2). Nucleotide sequence polymorphism analysis and a neutrality test for global PvLDH (*n* = 100) and PfLDH (*n* = 334) population were estimated using DnaSP ver. 5.10.00 [20] and MEGA4 program [21] as described above. To investigate the genetic relationships among global PfLDH or PvLDH haplotypes, the haplotype network for global PfLDH (*n* = 334) and global PvLDH (*n* = 100) was separately analysed using NETWORK ver. 5.0.0.3 with the Median Joining algorithm [25].

### Homology modelling and mutant analysis of PvLDH and PfLDH

The structural effects caused by amino acid changes identified in global PfLDH and PvLDH were analysed. The default protocol in the YASARA Structure ver.18.4.24 [26] was applied to construct three-dimensional (3D) homology models of PvLDH and PfLDH using the ‘hm_build.mcr’ macro file (http://www.yasara.org/hm_build.mcr). To construct homology models, PSI-BLAST [27] was conducted against PDB entries [28]. The homology models were built based on each template and then were refined using high-resolution energy minimization of the YASARA force field [29]. The best homology model based on the PfLDH template (PDB code: 1T2D) [30] was chosen among the reliable models. The characteristics of the model were evaluated using a Ramachandran plot [31], ERRAT [32] and ProSA [33]. The crystal structure of the PfLDH model (PDB code: 1T2D) [30] was retrieved from the RCSB Protein Data Bank [34]. To determine the impact of mutations on the structural stability, thermodynamic changes in wild-type and mutant structures of PvLDH and PfLDH were predicted using SDM ver. 2 [35] by estimating a stability score. All mutant models were generated using Andante [36] as implemented in SDM ver. 2. CUPSAT [37] and MAESTROweb [38] and were also used to improve the overall prediction accuracy of mutations under consideration. All structural graphics were visualized using an open-source code of PyMOL (The PyMOL Molecular Graphics System, ver. 1.7.2.1, Schrödinger, LLC) in Linux. Structural superpositions onto the wild-type structure were performed and visualized using the ‘cealign’ method script (https://pymolwiki.org/index.php/Cealign) in PyMOL v1.7.2.1.

## Results

### Genetic polymorphisms of Myanmar PvLDH

**Fig. 1:**
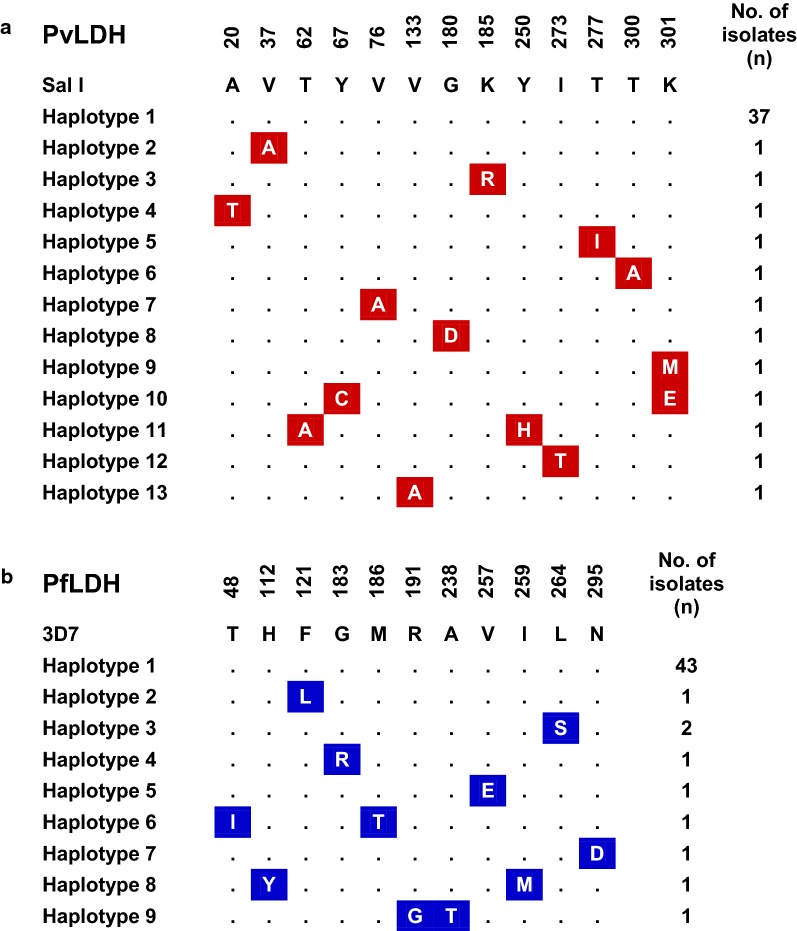
Amino acid sequence polymorphisms in Myanmar PvLDH and PfLDH. **a** PvLDH. Compared to the sequence of Sal I PvLDH (GenBank ID: XM_001615570), amino acid changes at 13 positions, including 12 dimorphic amino acid changes and 1 trimorphic amino acid change were identified in Myanmar PvLDH sequences. **b** PfLDH. Compared to the sequence of 3D7 PfLDH sequence (GenBank ID: XM_001349953), dimorphic amino acid changes at 11 positions were identified in Myanmar PfLDH sequences

A total of 49 PvLDH sequences were obtained from Myanmar *P. vivax* isolates. All of the sequences contained 951 nucleotides encoding 316 amino acids, indicating no insertion or deletion existed in the analysed sequences. When the sequences were compared with Sal I PvLDH (GenBank ID: XM_001615570), 45 single nucleotide polymorphisms (SNPs) were identified at 29 positions. Thirty-one of the SNPs were synonymous, and the other 14 were non-synonymous. The non-synonymous SNPs resulted in amino acid changes at 13 positions in Myanmar PvLDH, including 12 di-morphic amino acid changes (A20T, V37A, T62A, Y67C, V76A, V133A, G180D, K185R, Y250H, I273T, T277I, and T300A) and 1 tri-morphic amino acid change (K301M/E). Based on these amino acid polymorphic patterns, Myanmar PvLDH was classified into 13 distinct haplotypes (haplotype 1 to 13) (Fig. 1a).

### Genetic polymorphisms of Myanmar PfLDH

Fifty-two Myanmar PfLDH sequences were obtained. Comparing those sequences with the reference 3D7 PfLDH sequence (GenBank ID: XM_001349953) revealed 18 SNPs at 17 positions. Of these, 12 SNPs were non-synonymous and resulted in di-morphic amino acid changes at 11 positions (Fig. 1b). No insertion or deletion mutations were found in the Myanmar PfLDH sequences. Myanmar PfLDH was classified into 9 different haplotypes (haplotype 1 to 9) based on these amino acid polymorphic patterns (Fig. 1b).

### Genetic diversity and neutrality of Myanmar PvLDH and PfLDH

**Fig. 2:**
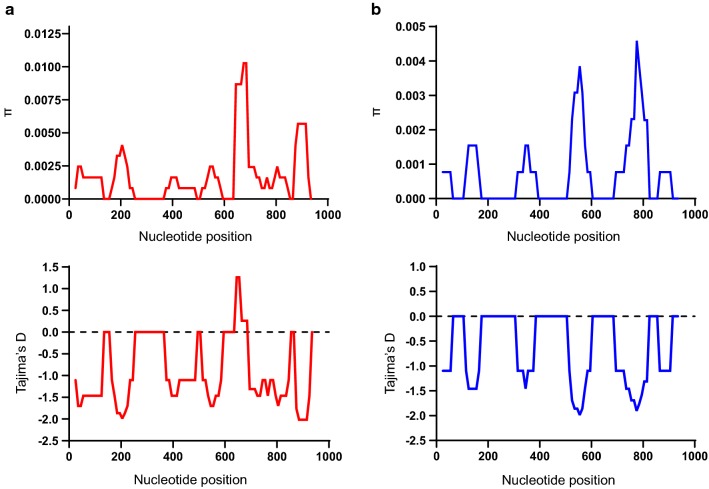
Nucleotide diversity and natural selection of Myanmar PvLDH and PfLDH. Sliding window plot analyses show nucleotide diversity (π) and Tajima’s D values across the Myanmar PvLDH (**a**) and PfLDH (**b**) sequences analysed in this study. A window size of 100 bp and a step size of 25 bp were used

Sequence analysis was performed to examine nucleotide diversity and neutrality of PvLDH and PfLDH among Myanmar *P. vivax* and *P. falciparum* population. The average number of pairwise nucleotide difference (*K*) for Myanmar PvLDH was 1.655 (Table 1). The overall haplotype diversity (Hd) and nucleotide diversity (π) of Myanmar PvLDH were estimated to be 0.767 ± 0.061 and 0.00174 ± 0.00022, respectively. Myanmar PvLDH was under negative selection as predicted by the negative values of Tajima’s D (− 2.481, *P* < 0.01), Fu and Li’s D (− 5.296, *P* < 0.02) and Fu and Li’s F (− 5.114, *P* < 0.02). The dN–dS value was − 0.004 ± 0.002. In the case of Myanmar PfLDH, the *K* and Hd values were 0.691 and 0.469 ± 0.087, respectively (Table 1). The π value was 0.00073 ± 0.00018. Myanmar PfLDH was also predicted to be under negative selection, which was supported by the negative values of Tajima’s D (− 2.533, *P* < 0.001), Fu and Li’s D (− 5.154, *P* < 0.02) and Fu and Li’s F (− 5.040, *P* < 0.02). The value of dN–dS was 0. These results suggest that both PvLDH and PfLDH were under negative purifying selections in Myanmar *P. vivax* and *P. falciparum* populations. To examine whether specific region(s) of each LDH region were under selection, a sliding window plot analysis was performed. Nucleotide diversity (π) was not evenly distributed throughout PvLDH and PfLDH of Myanmar *P. vivax* and *P. falciparum* populations. Higher nucleotide diversity was found in the C-terminal regions of both PvLDH and PfLDH (Fig. 2). Sliding window analysis of Tajima’s D values also showed similar patterns in PvLDH and PfLDH.

**Table 1:** Estimates of DNA sequence polymorphism and tests of neutrality of PvLDH and PfLDH in Myanmar *P. vivax* and *P. falciparum* isolates

	Segregating sites (S)	Singleton variable sites	*K*	H	Hd ± SD	π ± SD	dN–dS ± SD	Tajima’s D (*P* value)	Fu and Li’s D (*P* value)	Fu and Li’s F (*P* value)
PvLDH (*n* = 49)	29	26	1.655	21	0.767 ± 0.061	0.00174 ± 0.00022	− 0.004 ± 0.002	− 2.481 (*P* < 0.01)	− 5.296 (*P* < 0.02)	− 5.114 (*P* < 0.02)
PfLDH (*n* = 52)	17	16	0.691	14	0.469 ± 0.087	0.00073 ± 0.00018	0	− 2.533 (*P* < 0.001)	− 5.154 (*P* < 0.02)	− 5.040 (*P* < 0.02)

S, Number of segregating sites; *K*, average number of nucleotide differences; H, number of haplotypes; Hd, haplotype diversity; π, observed average pairwise nucleotide diversity; dN, rate of non-synonymous mutations; dS, rate of synonymous mutations

### Genetic polymorphism of PvLDH in the global *P. vivax* population

**Fig. 3:**
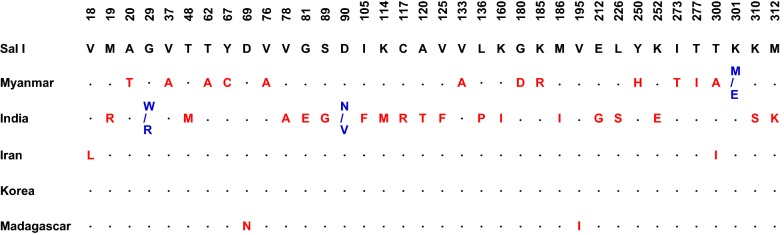
Amino acid sequence polymorphisms in global PvLDH. A total of 100 global PvLDH sequences were compared to the Sal I PvLDH reference sequence (GenBank ID: XM_001615570). A total of 36 amino acid changes, including 33 di-morphic amino acid changes and 3 tri-morphic amino acid changes, were identified among global PvLDH sequences

Amino acid polymorphism patterns in global PvLDH (*n* = 100) were analysed. Compared to Sal I PvLDH (GenBank ID: XM_001615570), a total of 39 amino acid changes at 36 positions were identified in the global PvLDH population (Fig. 3). However, these amino acid changes were not commonly identified in global PvLDH, rather they differed by country. The amino acid changes found in Myanmar PvLDH were not identified in PvLDH sequences from other countries including India, Iran, Korea and Madagascar. Similarly, amino acid changes found in other countries were country-specific. Most amino acid polymorphisms were di-morphic except for three amino acid changes that were tri-morphic (K301M/E in Myanmar PvLDH and G29W/R and D90N/V in India PvLDH). All the amino acid changes were observed at low frequencies ranging from 1 to 2% in the global PvLDH population.

### Genetic polymorphism of PfLDH in the global *P. falciparum* population

**Fig. 4:**
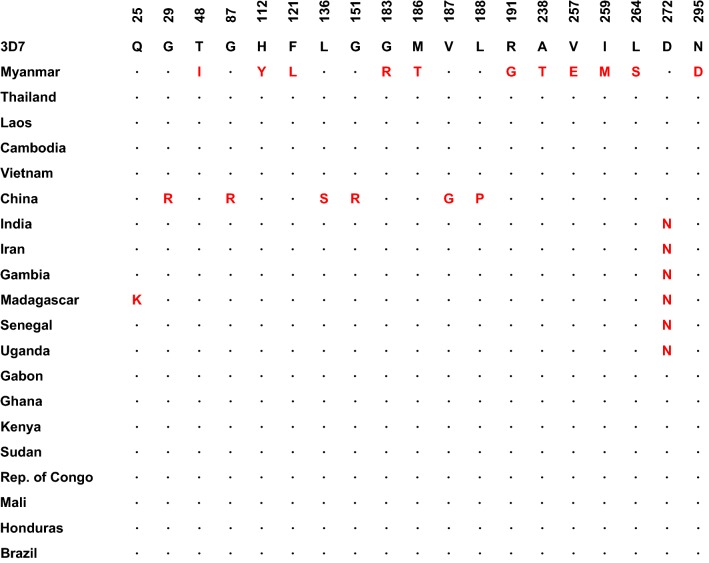
Amino acid sequence polymorphisms in global PfLDH. A total of 334 global PfLDH sequences were compared to the 3D7 PfLDH reference sequence (GenBank ID: XM_001349953). A total of 19 amino acid changes, all of which were di-morphic amino acid changes, were identified in global PfLDH sequences

Amino acid polymorphism patterns in global PfLDH (*n* = 334) were analysed. Compared to 3D7 PfLDH (GenBank ID: XM_001349953), a total of 19 amino acid polymorphisms, all di-morphic, were identified at 19 positions (Fig. 4). Similar to global PvLDH, global PfLDH showed different patterns of amino acid changes by country. Most amino acid changes (17 of 19) were identified in Myanmar and China PfLDH. Only one amino acid change (D272N) was commonly observed in isolates from several countries including India, Iran, Gambia, Madagascar, Senegal and Uganda PfLDH. No amino acid change was observed in PfLDH from Gabon, Ghana, Kenya, Sudan, Republic of Congo, Mali, Honduras, and Brazil. The overall frequencies of the amino acid substitutions in the global PfLDH population were low and ranged from 0.3 to 3.3%.

### Genetic diversity and neutrality of global PvLDH and PfLDH

**Fig. 5:**
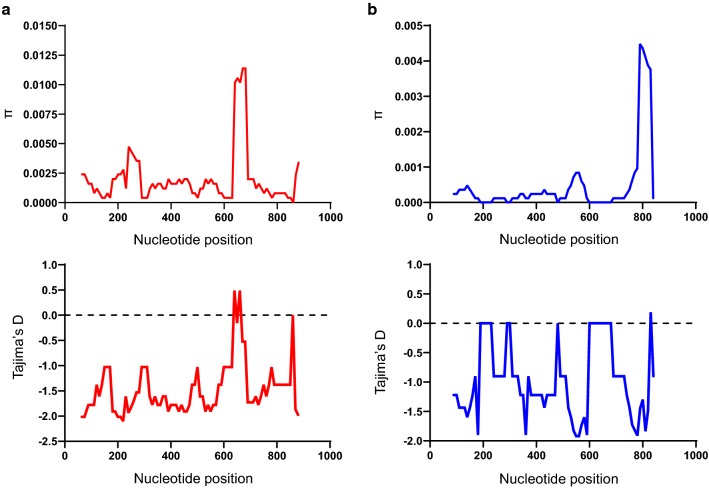
Nucleotide diversity and natural selection of global PvLDH and PfLDH. **a** PvLDH. Sliding window plot analyses show nucleotide diversity (π) and Tajima’s D values across global PvLDH sequences. A window size of 100 bp and a step size of 25 bp were used. **b** PfLDH. Sliding window plot analyses show nucleotide diversity (π) and Tajima’s D values across global PfLDH sequences. A window size of 100 bp and a step size of 25 bp were used

**Fig. 6:**
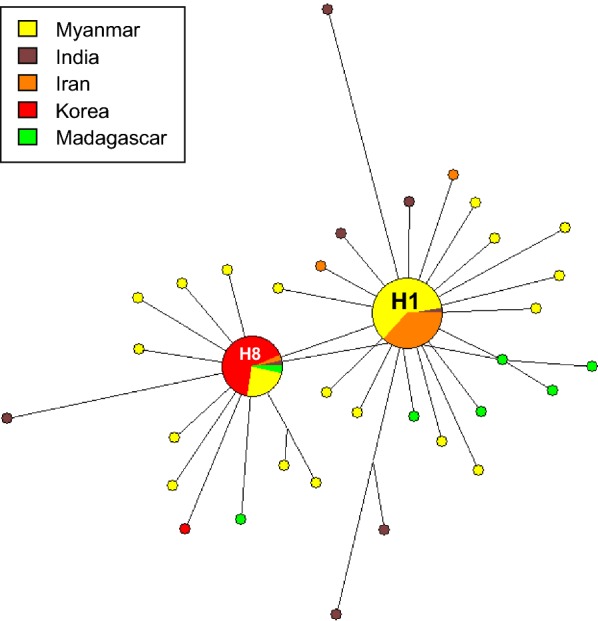
Haplotype network analysis of global PvLDH. A haplotype network of global PvLDH was constructed using the NETWORK program ver. 5.0.0.3 with the Median Joining algorithm. A network analysis of 100 sequences shows 35 distinct haplotypes. Branch lengths represent the proportion of divergence. The size of each node indicates the proportion of the total haplotype frequencies. The color of each node corresponds to a different geographic region

**Fig. 7:**
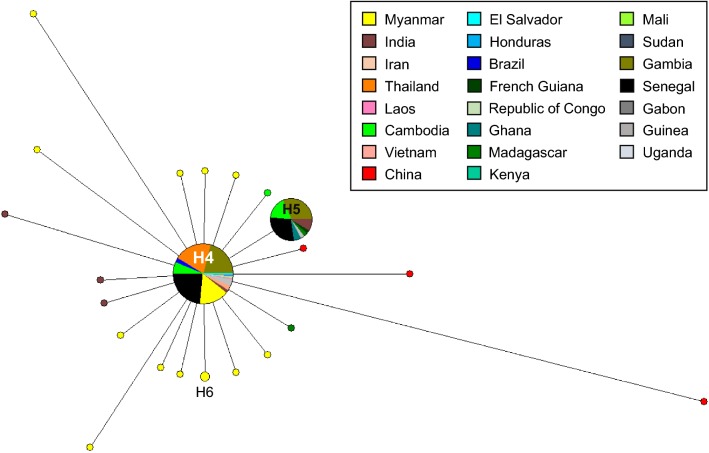
Haplotype network analysis of global PfLDH. Haplotype network of global PfLDH was constructed using the NETWORK program ver. 5.0.0.3 with the Median Joining algorithm. A network analysis of 334 sequences shows 22 individual haplotypes. Branch lengths represent the proportion of divergence. The size of each node indicates the proportion of the total haplotype frequencies. The color of each node corresponds to a different geographic region

To further examine whether natural selection had contributed to PvLDH and PfLDH diversity within the global *P. vivax* and *P. falciparum* populations, publicly available sequences deposited in the NCBI and PlasmoDB databases were analysed (Table 2). The dN–dS values for global PvLDH and PfLDH were 0 and –0.004 ± 0.002, respectively, indicating both genes were under strict purifying selections. In order to more closely explore the effects of natural selection on each PvLDH and PfLDH, the values for Tajima’s D, Fu and Li’s D and Fu and Li’s F were analysed. The Tajima’s D values for global PvLDH and PfLDH were − 2.26226 (*P* < 0.01) and − 2.28117 (*P* < 0.01), respectively. The values for Fu and Li’s D and Fu and Li’s F for global PvLDH and PfLDH were also negative, indicating both genes had been influenced by negative natural selection (Table 2). Sliding window plot analyses of nucleotide diversity (π) and Tajima’s D of the global PvLDH and PfLDH also revealed low genetic diversity and negative selection (Fig. 5). A haplotype network analysis of global PvLDH (*n* = 100) showed 35 closely linked haplotypes (Fig. 6). Two major haplotypes (H1 and H8) were identified, and the other haplotypes were singletons arising from H1 to H8. The most prevalent haplotype was haplotype 1 (H1), which shared the same nucleotide sequences as Sal I (GenBank ID: XM_001615570) with a frequency of 38.0%. The frequency of haplotype 8 (H8) was 29.0%. A haplotype network analysis of global PfLDH (*n* = 334) revealed a total of 22 distinct haplotypes (Fig. 7). The most prevalent haplotype was H4, which had the identical nucleotide sequences with 3D7 (GenBank ID: XM_001349953) and was shared by populations from different countries with a frequency of 83.2%. Haplotype 5 (H5) was the second most prevalent haplotype (frequency of 10.4%) and was mainly comprised of African PfLDH. Nineteen haplotypes, except for haplotype 6 (H6), were singletons. These haplotypes were linked to the most frequent H4 by short or long branches. Similar to global PvLDH, haplotype network analysis of global PfLDH revealed a simple cluster with a small number of haplotypes having low frequencies separated by short branches.

**Table 2:** Estimates of DNA sequence polymorphism and tests of neutrality of PvLDH and PfLDH in the global *P. vivax* and *P. falciparum* isolates

	Segregating sites (S)	Singleton variable sites	*K*	H	Hd ± SD	π ± SD	dN–dS ± SD	Tajima’s D (*P* value)	Fu and Li’s D (*P* value)	Fu and Li’s F (*P* value)
PvLDH (*n* = 100)	59	53	1.804	35	0.776 ± 0.032	0.0021 ± 0.0003	0	− 2.714 (*P* < 0.001)	− 8.118 (*P* < 0.02)	− 7.073 (*P* < 0.02)
PfLDH (*n* = 334)	31	29	0.374	22	0.297 ± 0.031	0.0005 ± 0.0001	− 0.004 ± 0.002	− 2.505 (*P* < 0.001)	− 10.126 (*P* < 0.02)	− 8.285 (*P* < 0.02)

S, Number of segregating sites; *K*, average number of nucleotide differences; H, number of haplotypes; Hd, haplotype diversity; π, observed average pairwise nucleotide diversity; dN, rate of non-synonymous mutations; dS, rate of synonymous mutations

### Structural impacts caused by amino acid changes in global isolates of PvLDH and PfLDH

**Fig. 8:**
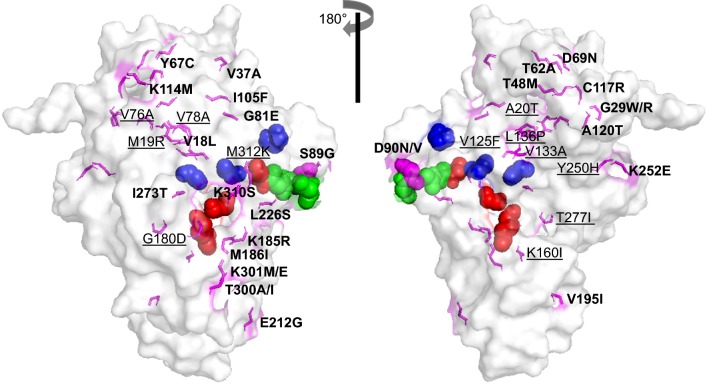
Polymorphism sites mapped to the 3D structure of PvLDH. Front and back views of the surface of PvLDH with mutated amino acid residues. The two views are rotated with respect to each other by 180° around the vertical axis. The 36 amino acid changes identified in global PvLDH are indicated with magenta sticks. The mutated residues, which were located on the external or internal regions, are depicted in bold or underlined, respectively. The functional residues of PvLDH are depicted as colored spheres: catalytic residues (R95, D155, R158 and H182) in red, the active site (K84) and cofactor-binding site (P235 and P239) in blue, and the substrate-specific loop (D90–N94) in green

**Fig. 9:**
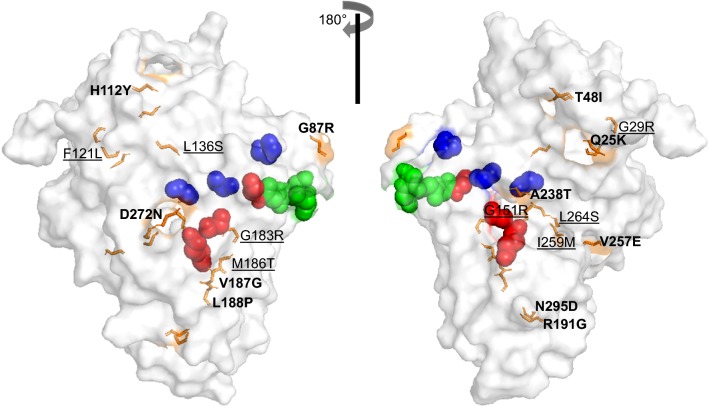
Polymorphism sites mapped to the 3D structure of PfLDH. Front and back views of the surface of PfLDH with mutated amino acid residues. The two views are rotated with respect to each other by 180° around the vertical axis. The 19 amino acid changes identified in global PfLDH are indicated with orange sticks. The mutation residues, which were located on the external or internal regions, were depicted in bold or underlined, respectively. The functional residues of PfLDH sre depicted as colored spheres: catalytic residues (R95, D155, R158 and H182) in red, the active site (K84) and cofactor-binding site (P235 and P239) in blue, and the substrate-specific loop (D90–N94) in green

The structural effects of amino acid changes found in global isolates of PvLDH and PfLDH were investigated. Several PvLDH crystal structures (PDB codes: 5HRU at 1.71 Å, 5HTO at 1.90 Å and 2A92 at 2.04 Å) were reported previously (Additional file 3: Table S3), but their resolutions were not high, Accuracy of the template structure such as resolution should be carefully considered to construct the most reliable homology model [39]. Therefore, a homology model of PvLDH was constructed based on a high-resolution crystal structure of PfLDH (PDB code: 1T2D at 1.1 Å resolution), due to the low-resolution crystal structures for PvLDH. The constructed PvLDH model had 94.1% of the amino acid residues in a favorable region according to the Ramachandran plot (Additional file 4: Fig. S1a), a ProSA Z-score of − 9.82 (Additional file 4: Fig. S1b) and an ERRAT overall quality score of 96.74 (Additional file 4: Fig. S1c). Superposition between PvLDH and PfLDH showed a root-mean-square deviation (RMSD) of 0.06, suggesting the two proteins shared the same overall folding pattern (Additional file 5: Fig. S2a). The models predicted a Rossmann fold in all pLDH enzymes and the NADH cofactor-binding site [40, 41] that forms a series of alternating 6 β-strands and 5 α-helices (Additional file 5: Fig. S2b). Furthermore, PvLDH and PfLDH shared identical amino acid sequences at catalytic residues (R95, D155, R158, and H182) [42], the active site (K84), cofactor binding sites (P235 and P239) [43], and the substrate-specific loop (DKEWN, amino acid positions 90–94) [44] (Additional file 5: Fig. S2a). When all the amino acid changes found in global PvLDH and PfLDH were applied to the models, it suggested that they were scattered throughout the structures (Figs. 8 and 9). The structural impact of all non‐synonymous amino acid substitutions of PvLDH and PfLDH were analysed based on the RMSD and the predicted ΔΔG (kcal/mol) values between the wild-type and mutants.

Superposition between PvLDH wild-type and 40 mutants generated an RMSD of 0.001–0.059 (Additional file 6: Table S4). PfLDH wild-type and 19 mutants generated an RMSD of 0.001–0.053 (Additional file 7: Table S5). These results suggest that individual amino acid changes may not induce significant structural changes in both PvLDH and PfLDH. To improve the accuracy of potential stability changes (ΔΔG, kcal/mol) with each amino acid change, three different predictors (SDM ver. 2 [35], CUPSAT [37], and MAESTROweb [38]) were applied, but their coincidence results were inconclusive (Additional files 6 and 7: Tables S4 and S5). Although D90V/N of PvLDH was located in the substrate-specific loop, it is predicted not to significantly affect structural stability (Additional file 6: Table S4). These results collectively suggest that all amino acid changes identified in global PvLDH and PfLDH are unlikely to cause significant structural changes in the proteins.

## Discussion

Analyses of genetic polymorphisms in Myanmar PvLDH and PfLDH revealed a low level of genetic diversity in Myanmar *P. vivax* and *P. falciparum* populations. Both genes showed synonymous SNPs and non-synonymous SNPs, which generated different haplotypes in each gene: 13 haplotypes in PvLDH and 9 haplotypes in PfLDH. Global PvLDH and PfLDH sequence analyses also suggested that amino acid changes caused by diverse non-synonymous SNPs were identified among the global PvLDH and PfLDH. However, the frequencies of the amino acid changes were not high, and they were not evenly distributed among the global population but were country-specific. Overall genetic diversity was greater in PvLDH than PfLDH. This is consistent with previous findings on the genetic diversity of PvLDH and PfLDH in other countries [15–17, 42, 45]. These results are also consistent with a previous report of comparative genomic analyses suggesting more polymorphic events were identified in the *P. vivax* genome than *P. falciparum* [46].

Neutrality tests of Myanmar PvLDH and PfLDH indicated the influence of a strong purifying selection on Myanmar PvLDH and PfLDH. The dN–dS values of Myanmar PvLDH and PfLDH did not show a significant departure from zero. The significant negative values of Tajima’s D, Fu and Li’s D, and Fu and Li’s F for both PvLDH and PfLDH were also in agreement with the dN–dS values. Similar patterns of purifying selection were also identified in global PvLDH and PfLDH [17], suggesting the genetic conservation of global PvLDH and PfLDH was mostly maintained by purifying selection. This is probably due to functional constraints. Haplotype network analyses of global PvLDH and PfLDH were also consistent with purifying selection, each with one or a few modal haplotypes separated by short branches.

A total of 39 amino acid changes at 36 positions were identified in the 100 global PvLDH sequences analysed in this study. Regarding PfLDH, 19 amino acid changes at 19 positions were observed in the 334 global sequences. The amino acid changes found in global PvLDH and PfLDH were not all located at essential residues, including catalytic residues (R95, D155, R158 and H182), the active site (K84), cofactor binding sites (P235 and P239) and the substrate-specific loop (DKEWN, amino acid positions 90–94) [47]. One exception to this is D90V/N, which was found in the substrate-specific loop of PvLDH. The mutation D90V/N, however, does not alter the protein’s structural stability since no prediction demonstrated a significant impact on protein stability. These results suggest that the amino acid changes in global PvLDH and PfLDH are unlikely to cause significant structural change that may disrupt the function of the enzymes. The 3D structure analyses also supported this notion by suggesting that no significant conformational change is likely to be caused by these amino acid variations.

Concerns have been raised about the genetic diversity found in pLDH and how it might influence the diagnostic efficacy of pLDH-based RDTs [48–50]. However, the results of this study suggest that the two genes were well-conserved in global populations even though low frequencies of amino acid changes were identified in the global population. It is unlikely that these polymorphic patterns identified in global PvLDH and PfLDH can influence the diagnostic performance of pLDH-based RDTs. In fact, pLDH-based RDTs performed well in filed performance assessments in India and other countries despite the occurrence of different genotypes [51–53]. Blood samples used in this study were also correctly diagnosed to each species by pLDH-based RDT kits (unpublished observations). These collectively suggest that epitopes of PvLDH and PfLDH targeted in RDTs are well-conserved in the global populations and therefore pLDH-based RDTs would be reliable to diagnose malaria in the field conditions. However, it would be noteworthy to mention that accuracy of RTDs can be affected by low parasitaemia and residual parasite antigens after clearance [4, 6, 54]. And therefore, continuous efforts to develop RDTs with higher sensitivity and specificity should be performed.

Functional and structural constraints of global PvLDH and PfLDH are also highly informative since pLDH is a promising drug target [55]. Plasmodial LDH is an essential enzyme in the central metabolic pathway of malaria parasites [14, 56–58]. Due to substantial differences in the human ortholog, pLDH is an attractive target for developing anti-malarial drugs [47, 59]. Inhibition of pLDH prevents adenosine triphosphate (ATP) production, which kills malaria parasites [60]. The results obtained in this study may help to identify or design effective inhibitors for anti-malarial drugs targeting pLDH.

The limitation of this study is that only limited numbers of Myanmar and global PvLDH and PfLDH sequences were included in the analysis. To better understand the polymorphic nature of PvLDH and PfLDH, further analysis of PvLDH and PfLDH variations in the global *P. vivax* and *P. falciparum* populations would be necessary.

## Conclusions

Low levels of genetic diversity, which may be affected by strong purifying selection, were identified in global PvLDH and PfLDH populations. Although non-synonymous SNPs that induce amino acid changes in global PvLDH and PfLDH were found, these amino acid changes were not commonly distributed in the global population, and their frequencies were low. Despite differences in the amino acid sequence, functionally important residues that maintain the structure and function of pLDH are well-conserved. These data collectively suggest that pLDH is a useful target molecule for RDT. Further examination of the genetic diversity of pLDH in diverse global *P. vivax* and *P. falciparum* populations with a larger number of isolates is necessary to better understand the polymorphic nature and evolutionary aspects of PvLDH and PfLDH.

## Supplementary information

**Additional file 1: Table S1.** Accession numbers of global PfLDH sequences.

**Additional file 2: Table S2.** Accession numbers of global PvLDH sequences.

**Additional file 3: Table S3.** The homology modeling templates (Top 40 hits).

**Additional file 4: Fig. S1.** Quality validation of the PvLDH homology models. (**a**) Ramachandran plot showing that most amino acid residues the residues (94.1%) are located in favored regions, allowed regions (5.1%), generously allowed regions (0.4%), and disallowed regions (0.4%). Red (A, B, L), yellow (a, b, l, p) and light yellow (~ a, ~ b, ~ l, ~ p) indicate the most favored regions, allowed regions, and generously allowed regions, respectively. White indicates disallowed regions. All non-glycine and non-proline residues are shown as closed black squares while glycines (non-end) are shown as closed black triangles. Disallowed residues are colored in red. (**b**) The ProSA energy profile [33] indicates a Z-score of − 9.82. (**c**) In the ERRAT plot [32], the overall quality factor is 96.74%.

**Additional file 5: Fig. S2.** Structural comparison between PvLDH and PfLDH. (**a**) Semi-transparent cartoon representations of PvLDH in cyan and PfLDH in orange reveal a similar overall fold with 0.06 RMSD. All functional residues are identical to the amino acids in the corresponding positions; catalytic residues (R95, D155, R158 and H182) in red spheres, the active site (K84) and cofactor-binding site (P235 and P239) in blue spheres, and the substrate-specific loop (D90–N94) in green spheres. (**b**) Perpendicular view of (**a**) shows a Rossmann fold-like subdomain, which is composed of 6 β-strands and 5 α-helices.

**Additional file 6: Table S4.** Potential changes of in the stability of PvLDH caused by 36 mutations using three different predictors.

**Additional file 7: Table S5.** Potential changes of in the stability of PfLDH caused by 19 mutations using three different predictors.

## Data Availability

The data supporting the conclusions of this article are provided within the article and its additional files. The original datasets analysed in this current study are available from the corresponding author upon request. The nucleotide sequences reported in this study have been deposited in the GenBank database under the accession numbers KU869730–KU869766 and KX885908–KX885922 for PfLDH and KU895512–KU895548 and KX885923–KX885934 for PvLDH.

## Abbreviations

dN: Rate of non-synonymous mutations
dS: Rate of synonymous mutations
H: Number of haplotypes
Hd: Haplotype diversity
*K*: Average number of nucleotide differences
LDH: Lactate dehydrogenase
PCR: Polymerase chain reaction
pLDH: Lactate dehydrogenase of *Plasmodium* spp
PvLDH: Lactate dehydrogenase of *Plasmodium vivax*
PfLDH: Lactate dehydrogenase of *Plasmodium falciparum*
π: Observed average pairwise nucleotide diversity
rRNA: 18S ribosomal RNA
RMSD: Root-mean-square deviation
S: Number of segregating sites
