# Supplementary figures and images for: Genetic diversity of Plasmodium vivax and Plasmodium falciparum lactate dehydrogenases in Myanmar isolates
Source: Malar J. 2020 Feb 4;19:60. doi: 10.1186/s12936-020-3134-y (PMC7001217; doi:10.1186/s12936-020-3134-y)

## Slide 1
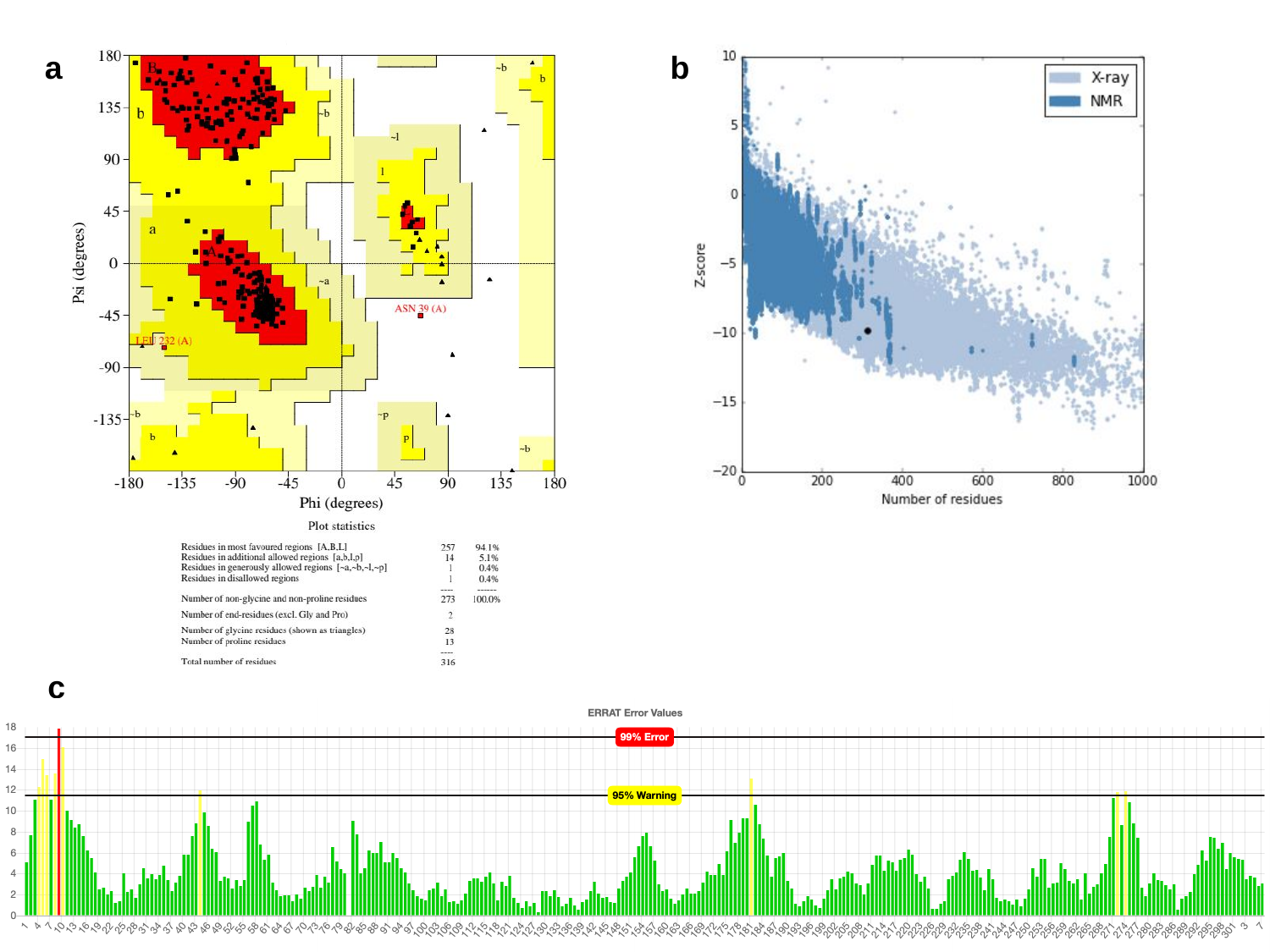

a
b
c

Supplement: Supplementary file 4 — Additional file 4: Fig. S1. Quality validation of the PvLDH homology models. (a) Ramachandran plot showing that most amino acid residues the residues (94.1%) are located in favored regions, allowed regions (5.1%), generously allowed regions (0.4%), and disallowed regions (0.4%). Red (A, B, L), yellow (a, b, l, p) and light yellow (~ a, ~ b, ~ l, ~ p) indicate the most favored regions, allowed regions, and generously allowed regions, respectively. White indicates disallowed regions. All non-glycine and non-proline residues are shown as closed black squares while glycines (non-end) are shown as closed black triangles. Disallowed residues are colored in red. (b) The ProSA energy profile [33] indicates a Z-score of − 9.82. (c) In the ERRAT plot [32], the overall quality factor is 96.74%. [file 12936_2020_3134_MOESM4_ESM.pptx]

## Slide 1
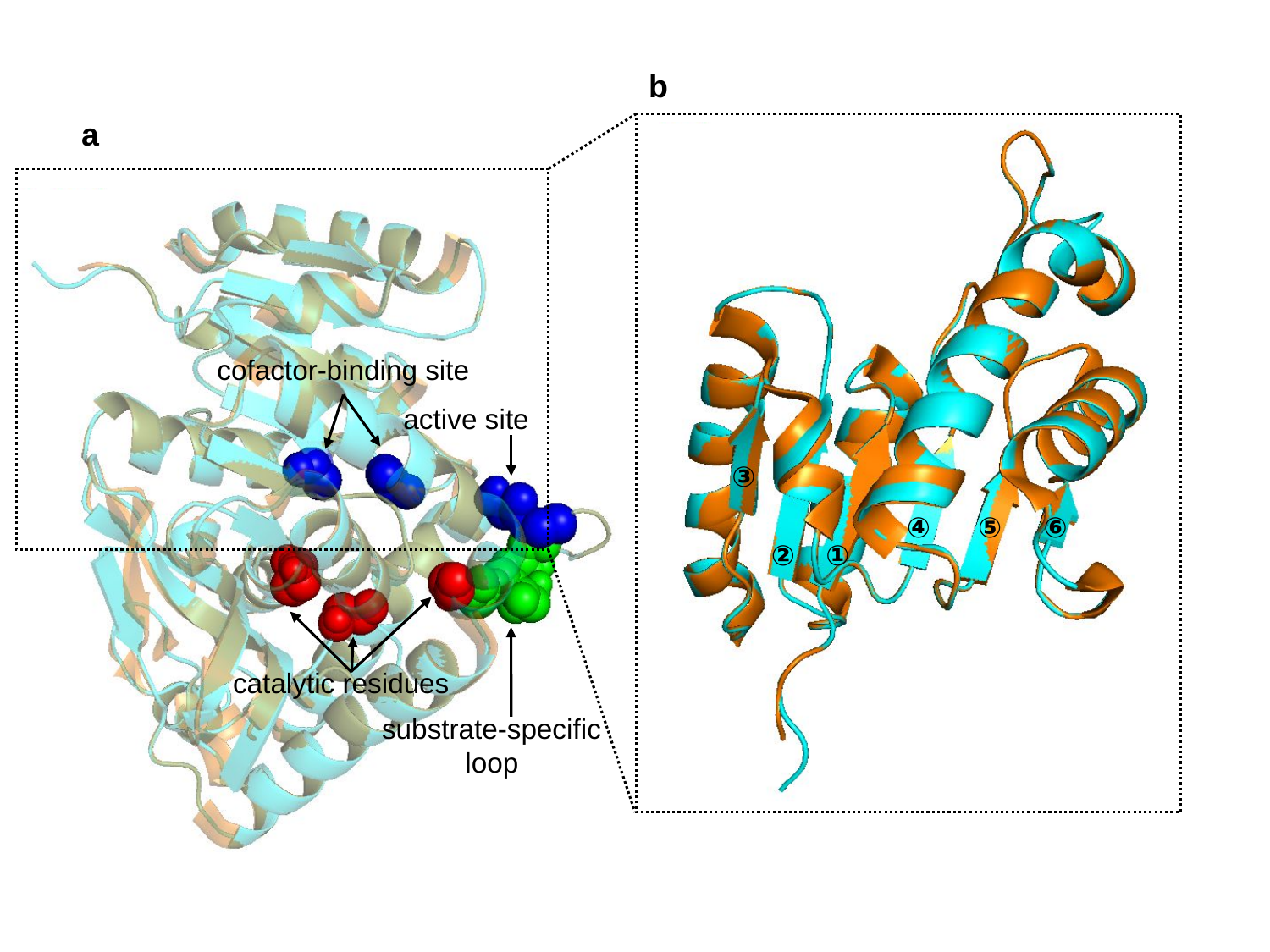

b
a
③
④
⑤
⑥
②
①
cofactor-binding site
active site
catalytic residues
substrate-specific
loop

Supplement: Supplementary file 5 — Additional file 5: Fig. S2. Structural comparison between PvLDH and PfLDH. (a) Semi-transparent cartoon representations of PvLDH in cyan and PfLDH in orange reveal a similar overall fold with 0.06 RMSD. All functional residues are identical to the amino acids in the corresponding positions; catalytic residues (R95, D155, R158 and H182) in red spheres, the active site (K84) and cofactor-binding site (P235 and P239) in blue spheres, and the substrate-specific loop (D90–N94) in green spheres. (b) Perpendicular view of (a) shows a Rossmann fold-like subdomain, which is composed of 6 β-strands and 5 α-helices. [file 12936_2020_3134_MOESM5_ESM.pptx]
